# Supervised multivariate analysis of sequence groups to identify specificity determining residues

**DOI:** 10.1186/1471-2105-8-135

**Published:** 2007-04-23

**Authors:** Iain M Wallace, Desmond G Higgins

**Affiliations:** 1 The Conway Institute of Biomolecular and Biomedical Research, University College Dublin, Belfield, Dublin 4, Ireland

## Abstract

**Background:**

Proteins that evolve from a common ancestor can change functionality over time, and it is important to be able identify residues that cause this change. In this paper we show how a supervised multivariate statistical method, Between Group Analysis (BGA), can be used to identify these residues from families of proteins with different substrate specifities using multiple sequence alignments.

**Results:**

We demonstrate the usefulness of this method on three different test cases. Two of these test cases, the Lactate/Malate dehydrogenase family and Nucleotidyl Cyclases, consist of two functional groups. The other family, Serine Proteases consists of three groups. BGA was used to analyse and visualise these three families using two different encoding schemes for the amino acids.

**Conclusion:**

This overall combination of methods in this paper is powerful and flexible while being computationally very fast and simple. BGA is especially useful because it can be used to analyse any number of functional classes. In the examples we used in this paper, we have only used 2 or 3 classes for demonstration purposes but any number can be used and visualised.

## Background

Proteins that evolve from a common ancestor can change functionality over time. For example, different related enzymes can bind to different substrates. Identifying the residues that cause these changes in specificity is important as this could allow one to alter the substrate specificity [1] or to predict the effect of mutations on the protein. The most common method for identifying SDP (specificity determining positions) starts with the construction of a multiple sequence alignment of the homologous proteins. Often the branching order of a phylogenetic tree exactly matches the known functional split between the proteins. Several methods have been used to identify the SDP's using only the tree and alignment [2,3], or combined with prediction of ancestral sequences [4] or with analysis of structures [5-7]. The evolutionary trace (ET) method, for example, identifies SDPs responsible for subgroup specificity by partitioning the tree or dendrogram into an increasing number of subgroups of similar sequences with subsequent analysis of their three-dimensional (3D) structures [5]

In cases when the functional split does not correspond to a clear phylogenetic split in the tree, other methods for identifying the SDPs have to be used. This situation can arise in highly divergent families, as proteins have multiple features that co-evolve along with specificity, such as the sub-cellar location or interaction with other proteins which may give a larger phylogenetic signal than the functional difference [8]. These methods normally require a multiple alignment, and a classification for the sequences.

Livingstone and Barton implemented a method called AMAPS that highlighted positions in an alignment that had different amino acid properties conserved between different sub-groups [9]. A similar idea is implemented in the Conserved Property Difference Locator method [10] which is available as a web server [11]. Mutual information has also been used as a measure to identify residues that confer specificity [12]. It is used to measure of how often a particular position in a sequence is conserved in one sub-family, and varies in another. Improvements were made to this algorithm which included taking into account the non-uniformity of amino acid substitutions via amino acid substitution matrices and a method for automatically setting cut-off thresholds [13]. The method of Hannenhalli and Russell [8] identifies the functional residues by comparing hidden Markov model profiles generated for each subgroup and calculating the relative entropy for each position. Positions with high relative entropy have very different amino acid distributions between the subgroups, and as such are considered possible SDPs. Pirovano et al. [14] showed that relative entropy exhibited undesirable asymptotic behaviour, and then reformulated it as "sequence harmony". Sequence logos [15] have also been used to visualise differences between two subfamilies [16,17].

A variety of multivariate statistical approaches have been applied to sequence analysis over the years. Principal coordinates analysis [18] was used to plot protein sequences in a low dimensional space that preserved the distances between them [19]. Later, principal component analysis (PCA) was used on multiple sequence alignments to identify possible functional residues [20]. The columns in the alignment were converted into a vector of binary variables of length 20, which represented the absence/presence of an amino acid at this position. PCA analysis of this matrix was then used to project the sequences onto 2 or 3 dimensions, which allowed identification of possible sequence clusters. The residue variables were also projected onto the same space, so that group specific residues could be identified. This method was implemented as a package called SequenceSpace. More recently this method has been automated so that the user does not have to manually identify the sequence groups [2] and made available as a web server [21]. Correspondence analysis (CA) has also been used to identify SDPs [22]. CA is used to decompose a chi-squared table between residues and sequences and has the attraction of automatically plotting both in the same space. The correspondences between residues and sequences (or groups of sequences) can then be seen from their proximity on the scatter plots.

CA and PCA are usually considered to be "unsupervised" techniques, to use the terminology of the field of machine learning and classification. This means that groups of objects that are of a-priori interest are not specified in advance. The axes are derived as those that account for maximum variance among the objects when these are plotted on them. Often, this is sufficient to obtain the information that one is interested in. The groups of interest may separate out in a useful manner along the first 2 or 3 axes or they may not; it will depend on the data set. Particular splits in the data may not show up in a useful manner on the plots. The most interesting splits can be masked by stronger trends of a less interesting nature such as amino acid composition. The analysis can be "supervised" by specifying groups and forcing the analysis to focus on these. In the case of Pazos et al [22], the relationships between the groups of sequences and residues are determined by an analysis of CA plots. Groups of sequences are represented as mean vectors and the residues that are closest to each vector are the ones that are most related to that group of sequences.

In this paper, we demonstrate the use of Between Group Analysis (BGA) for identifying SDPs from a sequence alignment. BGA is a supervised multivariate analysis method for sample discrimination and class prediction [23] and has been recently used to identify genes of interest from microarray data sets [24]. BGA is supervised by labelling each object (sequence) in advance as belonging to one of a small number of groups and forcing the axes to be those that split these groups as much as possible. Technically, the analysis is accomplished by finding those axes that maximise the between group variances. Informally, this is accomplished by treating the group centroids as the objects to be analysed and by carrying out a CA or PCA on these centroids. This produces results that are similar in appearance to those derived from multiple discriminant analysis but with the bonus of being robust when the number of variables exceeds the number of objects. BGA carried out using PCA is very similar to partial least squares discriminant analysis (PLS DA). In practice, BGA is carried out in two stages. These are automatically done using the MADE4 package [25]. Firstly, an ordination is computed using either PCA or CA on a data set in which the different groups are defined. BGA then finds linear combinations of the axes that maximise between-group variances and minimise within-group variances. This combination is very flexible as it can be used to examine any number of pre-specified groups. It has a further advantage in that it can be combined with a variety of data types from binary to continuous because of the way in which it can use PCA or CA.

## Results

### Lactate/Malate dehydrogenases

The results for the Lactate/Malate dehydrogenase case using the two different amino acid encoding schemes are shown in Figure 4. Each set of results is displayed using three vertical lines, each of which is a view of the single axis of the analysis. The sequences are plotted on the line on the left, the group centroids are in the middle and the variables/residues are plotted on the right. Each sequence is joined, by a line, to its group centroid. The ten most extreme variables at each end of the axis are displayed in the same order as they appear on the axis. In both sets of results, the variables at the top of the axis are most associated with the MDH group of sequences while the residues at the bottom are most associated with the LDH group.

**Figure 1:**
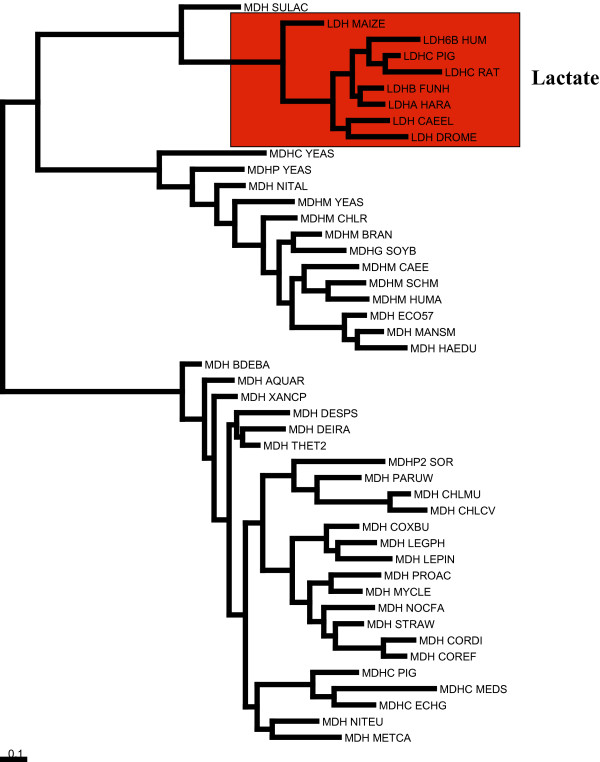
Phylogenetic tree of lactate/malate dehydrogenases sequences. The Lactate sequences are coloured in red.

**Figure 2:**
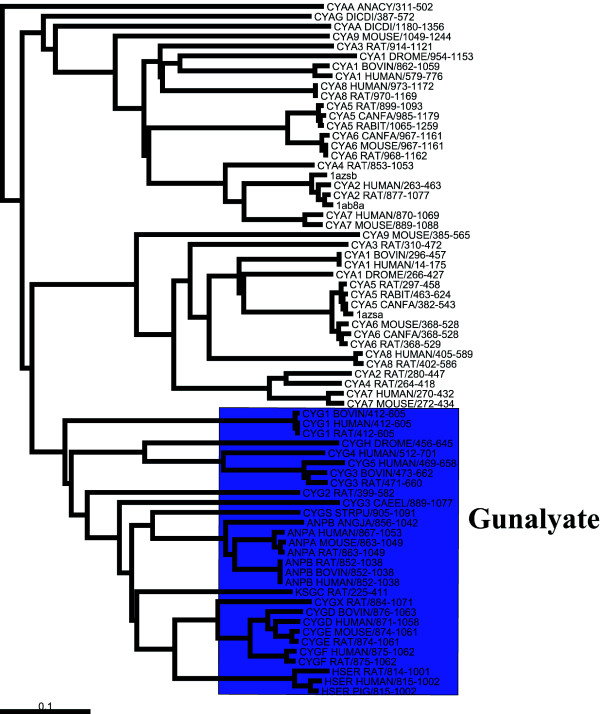
Phylogenetic tree of the nucleotidyl cyclases sequences. The guanylate sequences are coloured in blue.

**Figure 3:**
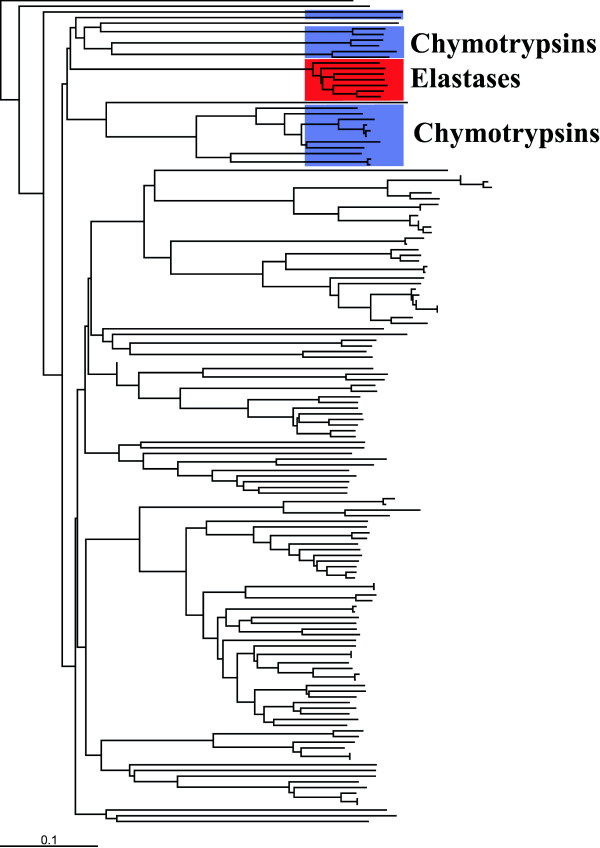
Phylogenetic tree of serine protease sequences. The elastases are highligthed in red and the chymotrypsins are in blue.

**Figure 4:**
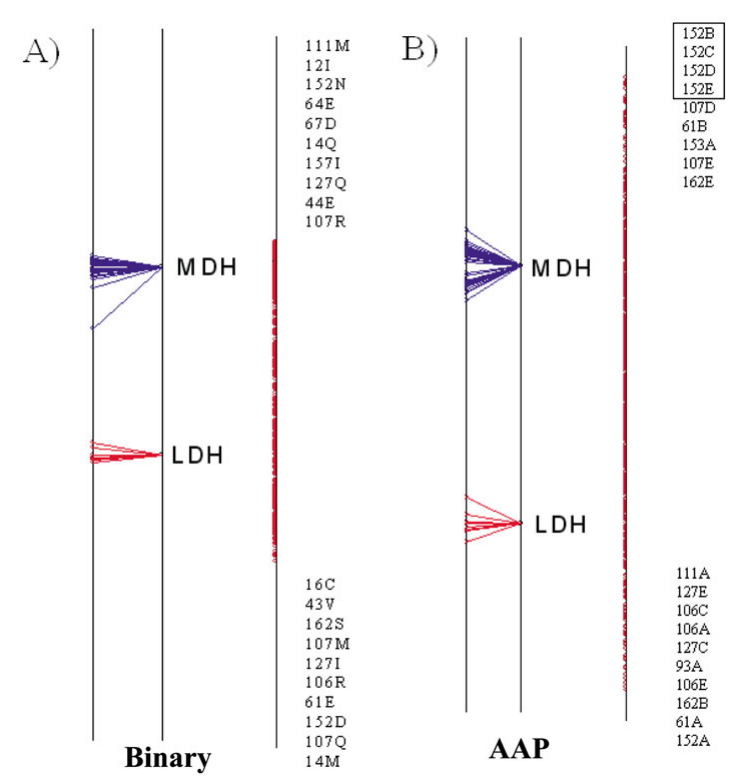
Axis 1 of the Between Group Analysis for the Lactate/Malate Dehydrogenase test case using the binary encoding (A) and the AAP encoding (B). In each example the sequence split is shown on the left, the residues are plotted on the right. The top 10 residues at either end of the axis are shown. Any residues that are plotted at the same coordinate are enclosed in a text box. Each variable consists of a number, which is the alignment position, followed by a residue type or factor, depending on which encoding system was used.

In both sets of results there is a very clear separation of the two groups of sequences. This is a useful indicator to tell if the method was able to successfully separate the two groups. The inter-group separation is much more than the intra-group separation. There are no obvious outliers in either analysis.

**Figure 5:**
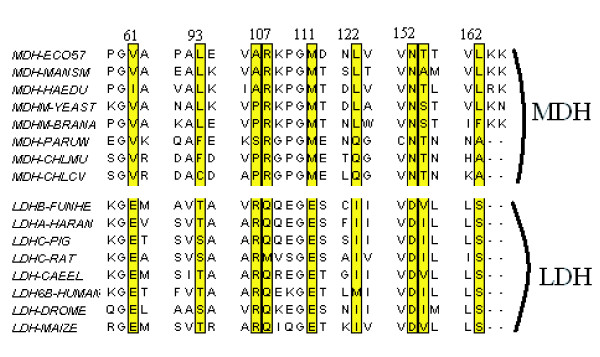
Alignment of a sample of the lactate/malate dehydrogenase sequences with positions highlighted that the analysis using the AAP residues identified as being important for specifity. The alignment was drawn with JalView [42].

Both sets of results correctly identify the Gln-Arg mutation as being important. This is position 107 in the alignment, which contains 7 Glutamine's and 1 Methionine in the Lactate set of sequences but only Arginine in the Malate set. It is the second most highly ranked position for the LDH group in the analysis with the binary representation, as well as the second position for the MDH group in the method using the AAP encoding. Figure 5 shows the positions, highlighted in the alignment, that were identified by the analysis using the AAP encoding.

The positions at the end of the axis are either strongly conserved in one group but not the other, or are strongly conserved in both groups but different from each other. If each group has a different strongly conserved residue the position will show up at both ends of the axis. This is true for the top position in the AAP encoding scheme, 152, which is a fully conserved D in the LDH group and N in MDH. The top position in the Binary encoding scheme is position 14, a fully conserved M in LDH and Q in MDH. Positions that are strongly conserved in one group and not the other will show up with a residue and position on the end of the axis corresponding to the group of sequences that they are conserved in, but there won't be a corresponding variable at the other end. One example of this is alignment position 106 which is an Arginine (R) in the lactate sequences but can be either A or P in the Malate case.

For this dataset, we compared our results with the SDPpred web server [26]. SDPpred predicted 15 residue positions as being important and these are shown in Table 2. 13 of these positions were also found in the top 10 residues at in the BGA analysis. The two positions that were predicted by SDPpred and not by BGA were position 9 and 21. Three positions were predicted by the BGA and not by SDPpred. They were E at position 64, D at 67 and E at position 44.

**Table 1:** The five factors for each of the amino acids as calculated by Altchely et al [28]

	**Factor A**	**Factor B**	**Factor C**	**Factor D**	**Factor E**
A	-0.591	-1.302	-0.733	1.57	-0.146
C	-1.343	0.465	-0.862	-1.020	-0.255
D	1.05	0.302	-3.656	-0.259	-3.242
E	1.357	-1.453	1.477	0.113	-0.837
F	-1.006	-0.590	1.891	-0.397	0.412
G	-0.384	1.652	1.33	1.045	2.064
H	0.336	-0.417	-1.673	-1.474	-0.078
I	-1.239	-0.547	2.131	0.393	0.816
K	1.831	-0.561	0.533	-0.277	1.648
L	-1.019	-0.987	-1.505	1.266	-0.912
M	-0.663	-1.524	2.219	-1.005	1.212
N	0.945	0.828	1.299	-0.169	0.933
P	0.189	2.081	-1.628	0.421	-1.392
Q	0.931	-0.179	-3.005	-0.503	-1.853
R	1.538	-0.055	1.502	0.44	2.897
S	-0.228	1.399	-4.760	0.67	-2.647
T	-0.032	0.326	2.213	0.908	1.313
V	-1.337	-0.279	-0.544	1.242	-1.262
W	-0.595	0.009	0.672	-2.128	-0.184
Y	0.26	0.83	3.097	-0.838	1.512

**Table 2:** Results from SDPpred web server [26] for the Lactate/Malate dehydrogenase sequences ranked by Z-Score

**Ranking**	**Position**	**Mutual Information**	**Z-Score**
1	152	4.44E-01	10.41
2	107	4.46E-01	8.44
3	61	4.61E-01	7.99
4	106	4.60E-01	7.13
5	16	4.43E-01	6.26
6	21	4.53E-01	6.19
7	157	3.67E-01	6.18
8	12	3.31E-01	6.18
9	14	4.56E-01	6.1
10	43	4.34E-01	6.05
11	127	4.40E-01	5.93
12	153	4.42E-01	5.63
13	111	3.54E-01	5.46
14	162	4.41E-01	5.23
15	9	3.87E-01	5.09

### Nucleotidyl cyclases

**Figure 6:**
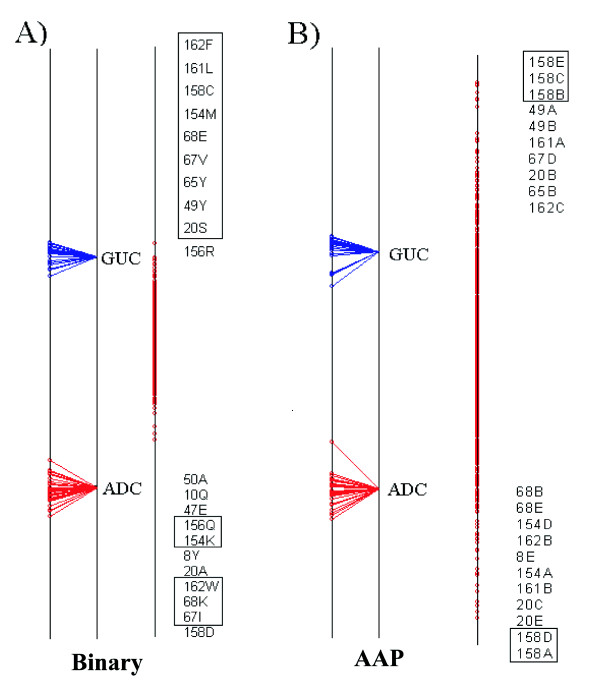
Axis 1 of the Between Group Analysis for the Nucleotidyl cyclases test case. Details as Figure 4

The results for the Nucleotidyl cyclases are shown in Figure 6, using the two different representation schemes. In both plots there is clear separation of guanylate cyclases (GUC) and adenylate cyclases (ADC).). The two positions (158 and 68), Glu-Lys (E->K) and Cys-Asp (C->D) that are sufficient to change the specificity are both identified by the method.

**Figure 7:**
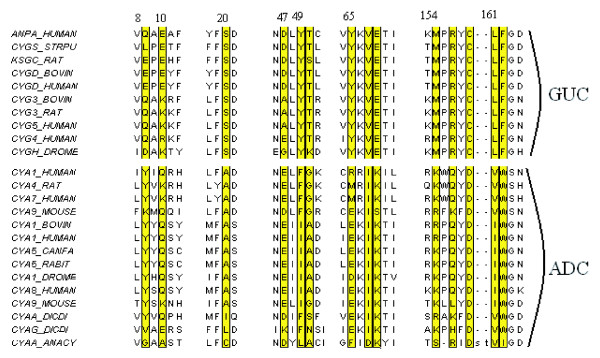
Alignment of a sample of Nucleotidyl cyclases sequences with positions highlighted that the analysis using the binary variables identified as being important for specifity. The alignment was drawn with JalView [42].

The analysis using the binary representation identifies all of the residues identified by Hannenhalli and Russell [8]. It also identified a position, 156 in the alignment, which only ever contains R in the GUC sequences, is mostly Q in the ADC but can be an R. This means that if there is a glutamine in that position it is likely to be an adenylate cyclase. Figure 7shows the positions, highlighted in the alignment, that were identified by the analysis using the binary encoding. The results were also compared with the SDPpred web server. The results are shown in Table 3. SDPpred predicted 5 positions as being important, all of which the BGA method identified.

**Table 3:** Results from SDPpred web server [26] for the Nucleotidyl cyclases sequences ranked by Z-Score.

**Ranking**	**Position**	**Mutual Information**	**Z-Score**
1	158	6.64E-01	19.87
2	162	6.49E-01	18.95
3	67	6.09E-01	18.25
4	68	6.38E-01	17.22
5	20	6.25E-01	16.63

### Serine Proteases

**Figure 8:**
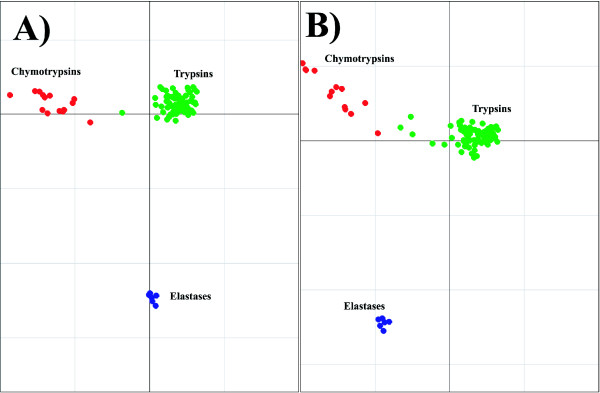
Demonstration of the effect of sequence weighting using the AAP encoding. The example using sequences weights is A). The unweighted example is B). The chymotrypsin sequences are plotted in red, trypsin sequences in green and the elastase are plotted in blue.

**Figure 9:**
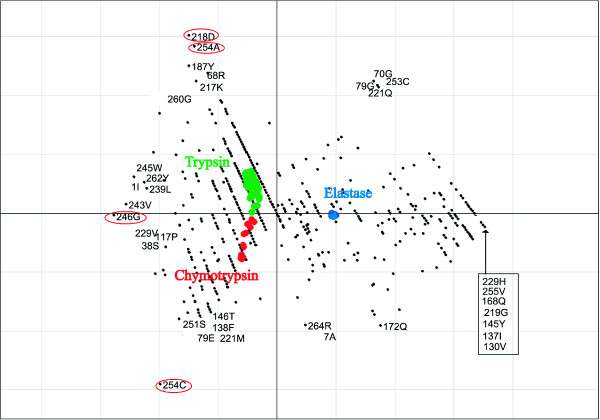
Axis 1 and 2 of the BGA results using CA for the serine protease alignment using the binary encoding showing both residues and sequences. Extreme residues are labelled. The trypsin sequences are plotted in green, chymotrypsin sequences in red and elastase sequences in blue, while residues are plotted in black. Positions that are thought to be in the binding pocket are circled in red.

Figure 8 demonstrates the effect of sequence weighting for the trypsin-like serine proteases. The unweighted results are shown in Figure 8B), while the analysis incorporating the sequence weighting is in Figure 8A). Axis 1 separates the trypsins from the chymotrypsins and the elastases. Axis 2 separates the chymotrypsins from the elastases. The effect is most noticeable for the chymotrypsins. The intra group separation for the chymotrypsins is smaller in the weighted example than the unweighted example. There is also a noticeable difference for the trypsin sequences. When no weighting is applied there are five trypsin sequences in the same half of the graph as the chymotrypsin group, while after applying the weights, there is only one. There is no noticeable difference in the two examples for the elastase sequences. The results for serine proteases using the binary representation are shown in Figure 9. As correspondence analysis was used as ordination technique for this analysis, both the sequences and the variables are plotted in the same space. The variables associated with a particular group of sequences are plotted in the same direction as the group centroid with the variables most associated with the group plotted furthest along this direction. Axis 1 of the CA separates the trypsin and the chymotrypsin sequences, and axis 2 separates the elastase sequences from trypsin and chymotrypsin sequences.

The most striking aspect of the results is that many residues are strongly associated with the elastase group. There are 7 residues plotted in the same position, furthest along axis 1. They are 130V, 137I, 145Y, 168Q, 219G, 229H, 255V, and they are all fully conserved for the elastases and not for the other two groups. The residue most associated with trypsin is D in position 218, which was also identified by Hannenhalli and Russell [8] as is the next residue, A in position 254. These two residues are defined as part of the binding pocket [27]. The other residue in the pocket, G, in position 246 in the alignment is located at the negative end of axis 1, on the opposite end of the elastase group. This is because it is column 246, in this alignment, for the elastase is blank, whereas the other two groups have a G in this position. This residue highlights the critical importance of the alignment for this type of analysis, as this position didn't show up as significant in the Hannenhalli and Russell [8] analysis. In the alignment used by Hannenhalli and Russell [8] this position isn't blank for the elastases. It in fact contains 50% of G, and as a result didn't show up as significant. Position 254 is also strongly associated with the chymotrypsin group, with a C at that position. The elastases have a fully conserved N in this position too, however this doesn't show up strongly in this analysis, as there is also an N present in one of the chymotrypsins.

**Figure 10:**
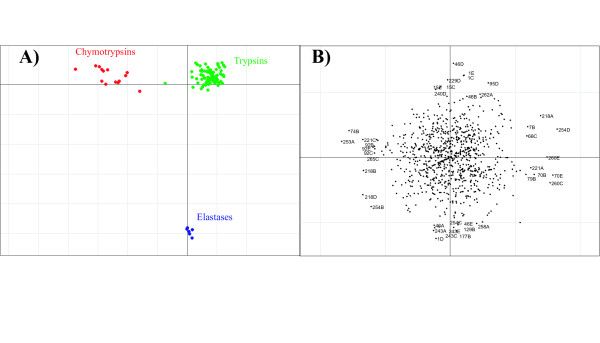
Axis 1 and axis 2 of the BGA results using PCA with the AAP encoding. Sequences are shown in A). Residues are shown in B).

Similar results can be seen when PCA is used as the ordination technique with the AAP encoding, as shown in Figure 10. Positions 218 and 254 strongly split the chymotrypsins and the trypsins. Interestingly, factor A at position 253 is strongly associated with the chymotrypsin group.

## Discussions and Conclusion

The inter relationship between amino acid residues and the functional properties of a protein is of great importance in understanding how that protein acts. Investigating how amino acids vary or are conserved at particular positions, depending on the function of the protein provides valuable insight into this relationship. One approach is to take a collection of sequences from homologous proteins and to represent them as a multiple alignment. This is followed by clustering the sequences into functional or phylogenetic groups and a search for residue/property correlations [2]. Unfortunately, it is then challenging to disentangle patterns of residue occurrence that are due to functional differences between the groups and patterns that are merely due to the process of amino acid substitution over time down different phylogenetic lineages. The fact that phylogenetic and functional groupings often disagree makes the situation even more difficult.

Multivariate analysis provides a logical alternative to traditional hierarchical clustering and has been used a number of times to analyse residue/property relationships [20,22]. This has proven to be useful for informal data exploration and visualisation. Most recently, Pazos et al [22] showed how to use Correspondence Analysis for this purpose, as an alternative to the more traditional PCA. They also showed how to analyse specific trends, of a priori interest, in the data. In this paper, we took this a stage further and showed how to use BGA as a general-purpose technique for protein alignment data exploration. We can combine it with CA in which case it becomes a way of carrying out a supervised CA, for any number of groups. With two groups, you get a single discriminating axis, which can be used like a discriminant function for classifying new sequences. This also ranks the variables (amino acids at each position) in terms of their discriminating power.

BGA can also be combined with PCA in which case there is a choice of how to encode the alignment information and measure amino acid occurrence in columns. Strict binary coding with CA enforces a strict view of presence or absence and is useful for emphasising clear trends in the data. It is, however, vulnerable to being misled by outlier amino acid occurrences. For example, if one sequence has one unusual amino acid in one position, the analysis will interpret this as potentially useful discriminating information. This is greatly improved by the use of pseudocounts. PCA has the advantage of allowing quantitative amino acid information to be used, such as amino acid physico-chemical properties [28]. In both case, sequence weighting is easy to apply to BGA using the MADE4 and ADE4 packages. This overall combination is powerful and flexible while being computationally very fast and simple. Finally, BGA is especially useful because it can be used to analyse any number of functional classes. In the examples we used in this paper, we have only used 2 or 3 classes for demonstration purposes but any number can be used and visualised.

In summary the method presented here is complementary to the other methods for identifying SDP's. It produces similar results, but provides an alternative method for viewing the results. This makes the method very suitable for data exploration.

## Methods

### Software

A combination of R packages from the Bioconductor package [29] were used to read in a sequence alignment, transform it into a data matrix, and to carry out the Between Group Analysis. They were:

#### ADE4

A general purpose data analysis package for ecological data sets [30]. It contains a very large variety of clustering, ordination and discriminant data analysis methods.

#### MADE4

A package that facilitates the analysis of microarray data using the ADE4 package [25]. It also includes graphical and visualisation tools which, for example, are used to display the results of a BGA analysis.

#### Seqinr

A package for reading different types of sequence files including Fasta and ClustalW format into R [31]

### Amino Acid Encoding Schemes

Two different encoding schemes were used to represent the multiple alignments for BGA.

#### Binary Encoding

Each column in the sequence alignment is represented by a binary vector of length 20 which represents the presence or absence of a particular amino acid at this position [20]. This results in a data matrix of dimension N×L×20 where N is the number of sequences and L is the length of the alignment. Pseudocounts can also be included.

#### Amino Acid Property (AAP) Encoding

Each residue in the alignment is represented by a vector of five continuous variables as given by Atchley et al [28]. They applied a multivariate statistic approach to reduce the information in 494 amino acid attributes into a set of five factors for each amino acid. These factors are shown in Table 1

Factor A is termed the polarity index. It correlates well with a large variety of descriptors including the number of hydrogen bond donors, polarity versus nonpolarity, and hydrophobicity versus hydrophilicity. Factor B is a secondary structure index. It represents the propensity of an amino acid to be in a particular type of secondary structure, such as a coil, turn or bend versus the frequency of it in an *α*-helix. Factor C is correlated with molecular size, volume and molecular weight. Factor D reflects the number of codons coding for an amino acid and amino acid composition. These attributes are related to various physical properties including refractivity and heat capacity. Factor E is related to the electrostatic charge.

Columns in the alignment containing more than 80% gaps were removed. When using the AAP encoding, the remaining gaps were replaced with the average value for the subfamily in that column.

PCA is the most suitable ordination technique for the AAP encoding as it can be used to ordinate data that contains continuous variables. CA, on the other hand, can only be used to ordinate data that contains positive integer values, such as the binary representation of a sequence alignment.

### Sequence Weights

A sequence-weighting scheme is useful to minimize the redundancy and emphasize the diversity each of the sub-family alignments. Position-based sequence weights [32] were calculated for the sequences in each of the sub-families and used as weights in the BGA calculation. These weights were used, as they are intuitive, easy to calculate and have previously been shown to perform well for profile searches.

The sequence weight is calculated using equation 1. The sequence weight is the sum of all the residue weights for a particular sequence. The residue weights are calculated by counting the number of different residue types (r) that are present at position i in the alignment, and the number of times that the particular residue in the sequence type appears (s). The inverse of the product of these two numbers is the residue weight. Unique residues in conserved columns are awarded the most weight.

Sequence Weight=∑i1(ri∗si)
 MathType@MTEF@5@5@+=feaafiart1ev1aaatCvAUfKttLearuWrP9MDH5MBPbIqV92AaeXatLxBI9gBaebbnrfifHhDYfgasaacH8akY=wiFfYdH8Gipec8Eeeu0xXdbba9frFj0=OqFfea0dXdd9vqai=hGuQ8kuc9pgc9s8qqaq=dirpe0xb9q8qiLsFr0=vr0=vr0dc8meaabaqaciaacaGaaeqabaqabeGadaaakeaacqWGtbWucqWGLbqzcqWGXbqCcqWG1bqDcqWGLbqzcqWGUbGBcqWGJbWycqWGLbqzcqqGGaaicqWGxbWvcqWGLbqzcqWGPbqAcqWGNbWzcqWGObaAcqWG0baDcqGH9aqpdaaeqbqaamaalaaabaGaeGymaedabaWaaeWaaeaacqWGYbGCdaWgaaWcbaGaemyAaKgabeaakiabgEHiQiabdohaZnaaBaaaleaacqWGPbqAaeqaaaGccaGLOaGaayzkaaaaaaWcbaGaemyAaKgabeqdcqGHris5aaaa@4E2D@

### Pseudocounts

In this analysis it was very useful to add the pseudocounts to the binary encoding. This helps reduce the impact of small sample sizes if one group has very few sequences. Without pseudocounts, if a residue, at one position, is present once in one group and not present at all in a second group of sequences then the analysis will conclude this is an important residue at this position.

Pseudocounts have been widely used in calculating position specific weight matrix scores (PSSM). Again, they have been found to be useful with small sample sizes. When there are very few sequences present more pseudocounts should be added, but when there are many sequences much fewer are needed. The pseudocount frequency, g_i_, for an amino acid i in a column of a subfamily alignment was calculated using the method of Henikoff and Henikoff [33] as shown in equation 2 where q_ij _is the amino acid pair substitution probability for amino acid i and j, f_i _is the observed frequency of amino acid i, N is the total number of residues in the column and r is the number of residue types in the column. Q_i _is the actual amino acid frequency of amino acid i in the column after adding in pseudocounts. This method uses the amount of residue diversity in a column, r, to determine how many pseudocounts, *β*, to add. Pseudocounts were only used with the Binary Encoding scheme.

gi=∑jfjPjqijPj=∑iqijQi=Nfi+βgiN+ββ=5∗r
 MathType@MTEF@5@5@+=feaafiart1ev1aaatCvAUfKttLearuWrP9MDH5MBPbIqV92AaeXatLxBI9gBaebbnrfifHhDYfgasaacH8akY=wiFfYdH8Gipec8Eeeu0xXdbba9frFj0=OqFfea0dXdd9vqai=hGuQ8kuc9pgc9s8qqaq=dirpe0xb9q8qiLsFr0=vr0=vr0dc8meaabaqaciaacaGaaeqabaqabeGadaaakeaafaqabeqaeaaaaeaacqWGNbWzdaWgaaWcbaGaemyAaKgabeaakiabg2da9maaqafabaWaaSaaaeaacqWGMbGzdaWgaaWcbaGaemOAaOgabeaaaOqaaiabdcfaqnaaBaaaleaacqWGQbGAaeqaaaaakiabdghaXnaaBaaaleaacqWGPbqAcqWGQbGAaeqaaaqaaiabdQgaQbqab0GaeyyeIuoaaOqaaiabdcfaqnaaBaaaleaacqWGQbGAaeqaaOGaeyypa0ZaaabuaeaacqWGXbqCdaWgaaWcbaGaemyAaKMaemOAaOgabeaaaeaacqWGPbqAaeqaniabggHiLdaakeaacqWGrbqudaWgaaWcbaGaemyAaKgabeaakiabg2da9maalaaabaGaemOta4KaemOzay2aaSbaaSqaaiabdMgaPbqabaGccqGHRaWkiiGacqWFYoGycqWGNbWzdaWgaaWcbaGaemyAaKgabeaaaOqaaiabd6eaojabgUcaRiab=j7aIbaaaeaacqWFYoGycqGH9aqpcqaI1aqncqGHxiIkcqWGYbGCaaaaaa@60A3@

### BGA

The BGA was carried out in R using the MADE4 package. Firstly, the alignment file was read into R using the seqinr commands. The sequence weights were then calculated, and the groups defined.

The sequences were converted into a matrix using either the binary or AAP encoding schemes. Columns with more than 80% gaps were excluded. For the AAP encoding the remaining gaps were filled in with the average of the subgroup. Pseudocounts were added to the matrix calculated with the binary encoding.

The matrix, group definition, and type of ordination technique were then passed to the BGA function in Made4. If this ordination technique is CA then the matrix is pre-processed by multiplication with the sequence weights, but if the ordination technique is PCA then the sequence weights are passed in as row weights. The results are plotted using the MADE4 function.

### Test Cases

Three different test cases were chosen to demonstrate the method. They are Lactate/Malate dehydrogenases, Nucleotidyl cyclases and Serine Proteases. They have been used as examples by Hannenhalli and Russell [8] and Pazos et al [22] as well as others.

#### Lactate/Malate dehydrogenases

Lactate/Malate dehydrogenases share a similar substrate-binding domain. PFAM accession number, PF00056, "Lactate/Malate dehydrogenases, NAD binding domain". They are highly divergent and as such it is difficult to distinguish between lactate and malate subtypes. A single mutation Gln-Arg (position 102 in pdb 9ldta) is known to switch specifity from lactate to malate [34]. This example has been used by Hannenhalli and Russell [8] and Pazos et al [22]. In this study the alignment generated by Pazos et al was obtained from their website [35]. In this alignment there are 35 malate and 8 lactate sequences. The phylogenetic tree of the Lactate/Malate dehydrogenase sequences used by Pazos et al is shown in Figure 1. There is no simple phylogenetic separation between the two groups of sequences. There is a group of malate sequences that are more closely related to the lactate sequences than the rest of the malate sequences.

#### Nucleotidyl cyclases

Nucleotidyl cyclases are a family of cytosolic proteins that catalyse the reaction that transforms a nucleotide triphosphate into a cyclic nucleotide monophosphate. The cyclases act on either guanylate (GUC) or adenylate (ADC). The alignment used in this example is the same one used by Hannenhalli and Russell [8] and contains 41 adenylate and 29 guanylate sequences. The phylogenetic tree in Figure 2 was calculated from the alignment using the Neighbor-Joining method [36] implemented in ClustalW [37]. The tree was rooted using the add_root programme supplied by Manolo Gouy. Two point mutations, Glu-Lys and Cys-Asp, are sufficient to change the specificity of the enzyme from GUC to ADC [38]. These are positions 938 and 1018 of the protein sequence corresponding to the 3D structure of adenylate cyclase, 1ab8 [39].

#### Serine Proteases

Trypsin-like serine proteases are a large family of enzymes that cleave peptide bonds [40]. They have similar catalytic mechanisms but have different preferences for the bonds that they preferentially cleave. Trypsins cleave C-terminal to the positively charged amino acid residues, arginine and lysine. Chymotrypsins cleave bond that are flanked by large aromatic residues. Elastases cleave peptide bonds that are next to small-uncharged amino acid residues. The difference in specifity is caused by changes in the binding pocket [27]. An aspartic acid found in trypsin (Asp189) is usually replaced by a small residue in chymotrypsins (Ser) and elastases (Gly). Glycine at positions 216 and 226 in trypsin (also in chymotrypsins) is usually a valine or threonine in elastases [8]. In this study all of the sequences with EC numbers corresponding to trypsin, chymotrypsin, and elastase were extracted from the full alignment of PF00089 from PFAM [41]. This consisted of 117 trypsins, 17 chymotrypsins, and 7 elastases and these were realigned using ClustalW. Figure 3 gives the tree for this alignment, which was calculated using ClustalW and the Neighbor-Joining method. The elastase sequences all group together, while there is a set of chymotrypsin sequences, which are group closer to trypsin sequences than other chymotrypsins

## Authors' contributions

IW implemented and tested the experiment while, DH conceived and designed the experiment.
